# Inducing and Administering Tregs to Treat Human Disease

**DOI:** 10.3389/fimmu.2015.00654

**Published:** 2016-01-22

**Authors:** Ana Luisa Perdigoto, Lucienne Chatenoud, Jeffrey A. Bluestone, Kevan C. Herold

**Affiliations:** ^1^Department of Immunobiology, Yale University, New Haven, CT, USA; ^2^Department of Internal Medicine, Yale University, New Haven, CT, USA; ^3^Université Paris Descartes, Sorbonne Paris Cité, F-75475, Paris, France; ^4^INSERM U1151, CNRS UMR 8253, Hôpital Necker-Enfants Malades, Paris, France; ^5^Diabetes Center, University of California San Francisco, San Francisco, CA, USA

**Keywords:** T regulatory cells, autoimmunity, immune tolerance, immune therapy, cellular therapy

## Abstract

Regulatory T cells (Tregs) control unwanted immune responses, including those that mediate tolerance to self as well as to foreign antigens. Their mechanisms of action include direct and indirect effects on effector T cells and important functions in tissue repair and homeostasis. Tregs express a number of cell surface markers and transcriptional factors that have been instrumental in defining their origins and potentially their function. A number of immune therapies, such as rapamycin, IL-2, and anti-T cell antibodies, are able to induce Tregs and are being tested for their efficacy in diverse clinical settings with exciting preliminary results. However, a balance exists with the use of some, such as IL-2, that may have effects on unwanted populations as well as promoting expansion and survival of Tregs requiring careful selection of dose for clinical use. The use of cell surface markers has enabled investigators to isolate and expand *ex vivo* Tregs more than 500-fold routinely. Clinical trials have begun, administering these expanded Tregs to patients as a means of suppressing autoimmune and alloimmune responses and potentially inducing immune tolerance. Studies in the future are likely to build on these initial technical achievements and use combinations of agents to improve the survival and functional capacity of Tregs.

## Introduction

In order to maintain sufficient diversity needed to eliminate foreign antigens, the immune system needs mechanisms to avoid responses to self and to maintain tolerance. Inadequate immune responses can result in life-threatening infections and tumor growth but left unchecked, activation of the immune system can result in autoimmunity, allergy, and organ transplant rejection. T cell-mediated self-tolerance is sustained via a number of checkpoints in the thymus and the periphery (1). Immune regulation mediated by dedicated subsets of T lymphocytes, termed regulatory T cells (Tregs), is one major mechanisms of peripheral tolerance. Autoimmune diseases, including insulin-dependent type 1 diabetes (T1DM), systemic lupus erythematosus, rheumatoid arthritis, multiple sclerosis, result from a breakdown in self-tolerance. The development of autoimmunity in patients with malignancies treated with agents blocking costimulation highlights the critical role of the balance between Treg and effector T cell responses to prevent or halt the progression of autoimmune diseases (2–4). The major goal of immune therapies in autoimmune disease is to re-establish this balance.

This explains the ever growing interest over the last decade for strategies potentiating the functional capacity of Tregs and for T cell therapy approaches using *ex vivo* expanded Tregs.

Regulatory T cells include distinct subsets of T lymphocytes derived as a distinct lineage from the thymus, initially termed natural Treg, expressing the FOXP3 transcription factor and also from the periphery, initially termed adaptive Treg and encompassing both FOXP3^+^ and FOXP3^−^ cells. One may, thus, distinguish FOXP3^+^ Tregs, FOXP3^−^ IL-10-dependent Tr1, and LAP^+^TGF-β-dependent Th3 cells (1). Th3 cells play an important role in oral tolerance primarily through secretion of TGF-β and suppression of Th1 and Th2 cells (5). T regulatory type 1 (Tr1) cells develop from conventional T cells when exposed to regulatory dendritic cells (DCs) and have been shown to suppress T cell and antigen-presenting cell (APC) responses mainly via an IL-10 and TGF-β dependent mechanism (6). However, the bulk of critical Treg data has been generated based on the activity and specificity of the FOXP3^+^ Treg subset that develops within the thymic environment and in some circumstances, following peripheral exposure to self-antigens. This review will focus on the biology of FOXP3^+^ Tregs, therapeutic efforts to enhance their function *in vivo* and cell therapy strategies using *ex vivo* expanded FOXP3^+^ Tregs.

## Biology of Regulatory T Cells

**Figure 1:**
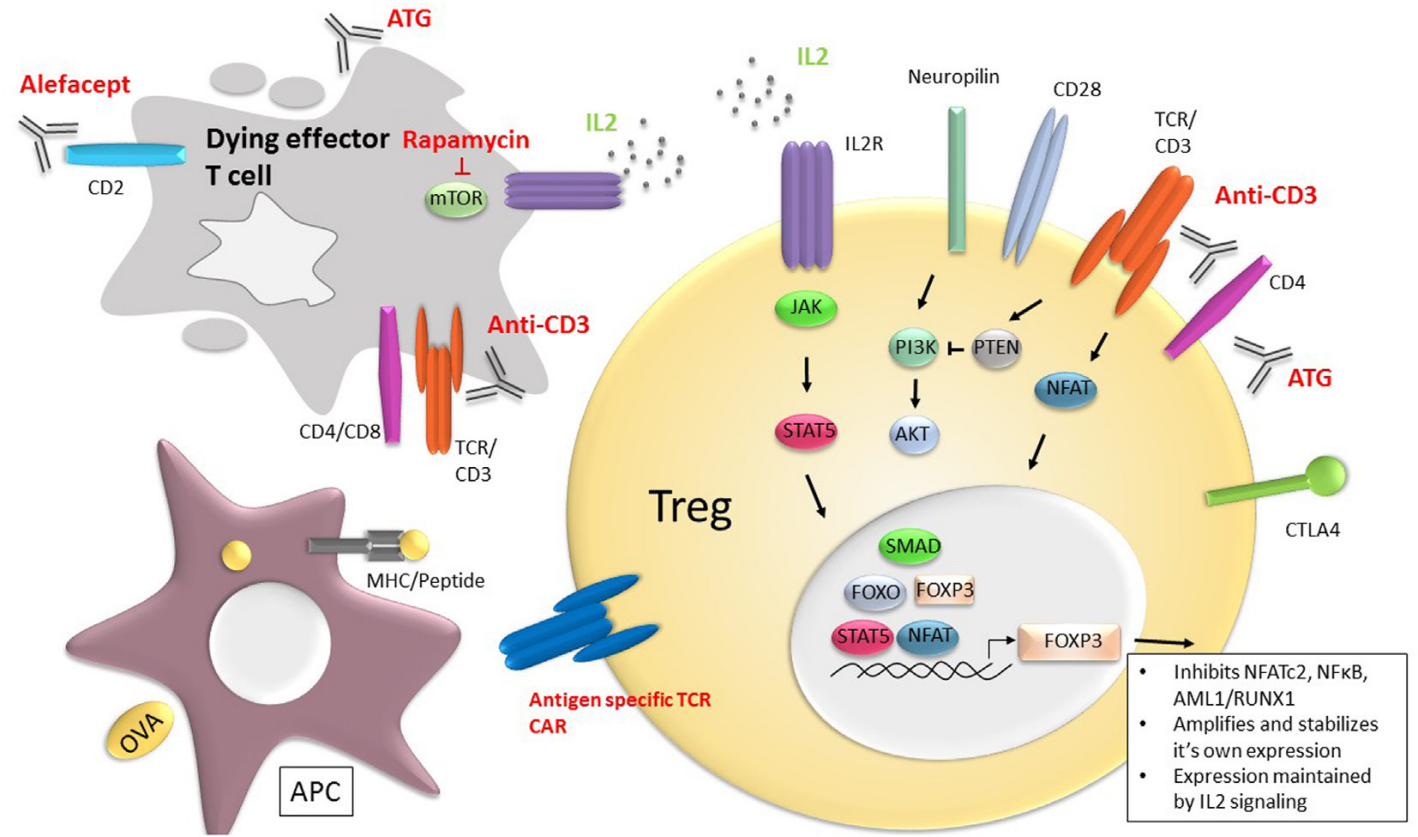
**Current and potential future therapies to promote Tregs and immune tolerance**. Therapies, such as rapamycin, anti-CD3 mAb, anti-thymocyte globulin (ATG), and Alefacept, a CD2 binding fusion molecule that eradicates CD2 expressing cells, exert their immune suppressive effect by eliminating effector T cells (gray cell) and tipping the balance in favor of Treg function and/or frequency. In addition, the anti-T cell receptor therapies, such as anti-CD3 mAbs may enhance survival and function of Tregs (yellow). IL-2, signaling through the ILR receptor (purple) and pSTAT5 (orange), is central to Treg survival and FOXP3 maintenance through the signaling cascades shown. FOXP3 inhibits cytokine gene expression by inhibiting NFATc2, as well as other transcriptional factors, such as NFκB and AML1/RUNX1. FOXP3 also facilitates Treg development by amplifying and stabilizing its own expression and inhibiting transcription factors required for other cell lineages, such as Tbet, GATA3, and RORγt. Cell-based therapies include use of Tregs engineered to express TCRs directed against specific antigens, including chimeric antigen receptors (CAR) (blue).

Regulatory T cells play a critical role in immune homeostasis and self-tolerance. They modulate immune responses by inhibiting effector T cells but they also serve an important function in the development and regulation of other lymphocyte and APC subsets. Tregs are found in both primary and secondary lymphoid organs as well as non-lymphoid tissues where they are thought to play a role in protection against immune damage as well as non-immune functions, such as tissue homeostasis and repair (7). Conventional Tregs are characterized by expression of the forkhead family transcription factor, Forkhead box P3 (FOXP3). FOXP3 was originally identified as playing a role in the development and maintenance of Tregs from observations in Scurfy mice, which develop a fatal lymphoproliferative disorder with CD4 T cell hyperactivation and production of proinflammatory cytokines (8). In humans, mutations in FOXP3 lead to a lack of functional Tregs and results in immunodysregulation polyendocrinopathy enteropathy X-linked (IPEX) syndrome that manifests as multi-organ autoimmunity, including diabetes, thyroiditis and allergy (i.e., eczema); in the absence of a bone marrow transplantation, death occurs within a year of birth (9–13). FOXP3, the lineage determinant of thymus-derived Tregs is a transcriptional repressor and inhibits cytoplasmic and calcineurin-dependent NFATc2, as well as other transcriptional factors, such as NFκB, and AML1/RUNX1 (Figure 1). Interaction of NFAT with FOXP3 is needed for suppressor function of Tregs. FOXP3 also facilitates Treg development by amplifying and stabilizing its own expression and inhibiting transcription factors required for other cell lineages, such as Tbet, GATA3, and RORγt (14).

The potency of Tregs lies in their ability to deploy various immunosuppressive mechanisms depending on the immunological context as well as extending their influence through the process of infectious tolerance (15, 16). Through contact-dependent mechanisms, Tregs have been shown to cause reduced T cell receptor (TCR)-induced calcium flux, NFAT, and NF-κB signaling and IL-2 production by effector T cells (17). In addition, by virtue of expression of CD25, they have been shown to consume IL-2, needed by effector T cells, and induce effector cell death by granzyme and perforin (18, 19). Tregs can inhibit T cell costimulation by either regulating CD80/86 expression on APCs through CTLA-4 or competing for CD28 binding (20–22). Finally, Tregs can produce regulatory cytokines, such as TGFβ, IL-35, and IL-10, which facilitate a key functional consequence of Tregs, namely bystander suppression (23–27).

The Treg TCR repertoire is highly skewed toward self-reactivity, which may be important in ensuring their ability to prevent the activation of autoreactive effectors and to avoid regulation of effector T cells needed for responses to pathogens and tumors. Work from our lab has shown that disease susceptibility loci, such as CTLA-4 and IL-2, in Type 1 diabetes lead to Treg instability (28). Immune effector T cells generated from destabilized FOXP3^+^ Tregs can mediate autoimmune reactivity, suggesting that some autoreactive effector T cells may have their origins in the Treg lineage. In addition, data suggest that a subset of effector T cells can become resistant to Tregs enhancing the potential activity of the autoreactive T cells in the type 1 diabetes setting (29).

## Definition and Role of Treg Subsets

Thymus-derived cells, tTregs [previously termed natural or nTreg (30)] are needed for general homeostasis and tissue repair. Tregs generated in the periphery, pTregs (previously termed adaptive or inducible), develop from conventional T cells (Tconv) and may be more important in controlling local auto-inflammatory responses (31). These subsets have distinct TCR repertoires where autoantigens may be presented differently in local tissues than during thymic development. In instances where the origin of Tregs is unclear, the general term “FOXP3^+^ Treg cell” is used (30). Although many markers have been identified as important in the immunobiology of Tregs, most are not unique to Tregs (Table 1). Signaling by IL-2 through the IL-2R has been shown to be particularly critical for Treg homeostasis and the maintenance of FOXP3 expression (32). CD127 expression is low on Tregs when compared to activated, conventional T cells, thus, the combination of CD4, CD25, and CD127 has been used to select T cells for functional studies as well as for expansion and adoptive immune therapy (33, 34). Other cell surface markers have been identified on Tregs that have functional significance. CD39 and CD73 are ectoenzymes that are involved in generation of adenosine, which inhibits effector T cells through interactions with the adenosine receptors A2AR/A2BR and in recruiting Tregs (35–37). Neuropilin-1 is a membrane-bound receptor on Tregs whose ligation by Semaphorin-4a restrains Akt phosphorylation and maintains Treg stability (38, 39). Helios, a transcription factor of the Ikaros family, was originally proposed as a marker of tTregs, distinguishing them from pTregs (40). However, a subsequent study showed that upon induction by APCs, pTregs can also express Helios, preventing its use as a tTreg marker (41). The cell surface receptors TIGIT and FCRL3 have also been recently identified as useful markers for Helios+ Tregs. TIGIT is associated with lineage stability and suppressive capacity. TIGIT+ Tregs were reported to selectively inhibit Th1 and Th17 but not Th2 T cell responses through an Fgl2-dependent mechanism (42–44). In addition, Smigiel et al. have discriminated Treg subsets into tissue-resident (eTregs: CD44^hi^CD62^lo/−^), which are dominant in non-lymphoid tissues and highly proliferative or central Tregs (CD44^lo^CD62L^+^), which are quiescent and recirculate through the secondary lymphoid tissues (45). The latter are dependent on IL-2 for survival and function, whereas the former are largely controlled by signaling through the TCR and IL-33, hence, their role in the control of antigen-specific responses that occur in peripheral tissues.

**Table 1:** **Markers of Tregs**.

	Marker	Ligand and interacting proteins	Function
Cell surface	CD4	MHCII	T cell marker and coreceptor for TCR
	TCR/CD3	Peptide/MHC	Required for Treg activation and suppressive function
	CTLA-4	CD80/CD86	Interacts with CD80 and CD86 on APCs to inhibit T cell activation through competition for costimulation of CD28. (20–22)
	CD28	CD80/CD86	Costimulatory signal required for differentiation of tTregs to eTregs. (21, 22)
	CD25 (IL-2R α chain)	CD122 and CD132 as part of IL2 receptor	IL-2 binding regulates Foxp3 expression, induces Treg proliferation, and is important for Treg survival (32, 46–52)
	CD127 (Interleukin 7 receptor α)	CD132 as part of IL7 receptor	Low CD127 expression compared to conventional T cells is characteristic of Tregs (33, 34)
	GITR	GITR-L	Seems to have positive effects on effector T cells and inhibitory effects via Tregs but role remains unclear (53, 54)
	Neuropilin	Plexin receptors, semaphorins	Highly expressed on tTregs and restrains Akt activation. Important in maintenance of Treg stability and has role in methylation (38, 39)
	TIGIT	CD155	Highly expressed on tTregs. Useful marker for Helios+ Tregs (42–44)
	FCRL3		Along with TIGIT, may help differentiate Helios+ from Helios− FOXP3 memory Tregs (44)
	OX40 (CD134)	OX40L	Inhibition of Treg suppressive function when stimulated (55, 56)
	CD45RA/RO	CD4/CD8 and TCR/CD3 complex	CD45RA is predominantly a naïve T cell subset and CD45RO a memory T-cell subset (57, 58)
	CD73 and CD39	Adenosine phosphates	Ectoenzymes that generate adenosine to inhibit effector T cell function. Role in Treg recruitment (35–37, 59)
	CD44	Hyaluronic acid, osteopontin and other ECM components	Increased expression in eTregs (45)
	CD62L	GlyCAM1, MadCAM1, CD34	Increased in thymic Tregs and functions as homing receptor (45)
	KLRG1	Cadherins	Expressed in a small number of peripheral Tregs and seems to represent a terminally differentiated Treg subset (60–62)
	ICOS	ICOS-L	Costimulatory receptor for TCR (63, 64)
Transcription factors	FOXP3		Master regulator essential for development, maintenance, and function of Tregs. Represses NFATc2, NFkB, AML1/RUNX1 (16)
	STAT5		Downstream of IL2 signaling. Stabilizes FOXP3 expression (65)
	NFAT		Positively regulates Foxp3 gene expression (66–68)
	AP-1		Positively regulates Foxp3 gene expression (68)
	Helios		Initially identified as a marker of tTregs, but more recently found on pTregs as well (40, 41, 44)
	SMAD3		Positively regulates Foxp3 gene expression (67, 69)
	IRF4		Role in differentiation of Tregs and is important for Treg function in adipose tissue (70–72)
	BLIMP-1		Role in differentiation of Tregs and is important for maintenance of transcriptional signature in eTregs (72)
	PPAR-γ		Role in VAT Tregs to reduce insulin resistance (73, 74)

*AP1, activation protein 1; APCs, antigen-producing cells; BLIMP1 or PR domain zinc finger protein 1 (PRDM1); CTLA-4, cytotoxic T-lymphocyte-associated protein 4; ECM, extracellular matrix; FCRL3, Fc receptor-like protein 3; FOXP3, forkhead box P3; GITR, glucocorticoid-induced TNFR-related protein; ICOS, inducible T cell costimulator; ICOS-L, ICOS ligand; IRF4, interferon regulatory factor 4; KLRG1, Killer cell lectin-like receptor subfamily G member 1; NFAT, nuclear factor of activated T-cells; PPAR-γ, peroxisome proliferator-activated receptor gamma; SMAD3, mothers against decapentaplegic homolog 3; STAT, signal transducer and activator of transcription; TCR, T cell receptor; TIGIT, T cell immunoreceptor with Ig and ITIM domains; VAT, visceral adipose tissue*.

PD1, implicated in negative regulation as well as a marker of T cell activation, is also expressed on Tregs. As in conventional cells, activation of PD1 on Tregs inhibited responses to anti-CD3 mAb but induced a different cluster of genes in Tregs compared to those activated in conventional T cells (75, 76). Finally, some investigators have subdivided FOXP3^+^ Tregs into effector/memory phenotypes in much the same manner as conventional T cells (57, 58, 77).

Studies in recent years have identified Treg populations in non-lymphoid tissues, including the skin, visceral adipose tissue (VAT), liver, intestine, skeletal muscle, bone, lungs, and placenta. Tissue-specific Tregs have unique phenotypes and roles depending on their location, varying with regard to frequency, TCR repertoire, cytokine production, chemokine receptor expression, and mechanism of action (15, 78–80). Immune-related functions in non-lymphoid tissues include suppression of T cell responses as well as limiting inflammation through control of myeloid populations (70, 73, 74, 78–80).Tregs are recruited by unique homing receptors to the skin and intestinal mucosa where they are needed to regulate the immune response against infectious pathogens as well as tolerance of commensal organisms (78). TGF-β and IL-10, produced by Tregs, play an important role in Treg function in the intestine and the intestinal microbiome also seems to influence Treg development and induction (81). Tregs in VAT express peroxisome proliferator-activated receptor gamma (PPAR-γ), a transcription factor that is required for the accumulation of Tregs at this location where they have a positive effect on insulin resistance and glucose metabolism (73, 74). VAT Tregs express IL-10 and TGF-β and depend on IRF4, BATF, and IL-33 (70).

There is growing evidence that Treg expansion occurs at sites of tissue injury and plays an important role in tissue repair and regeneration beyond immune regulation by influencing tissue resident non-immune cells. In injured skeletal muscle, a Treg population was identified that can promote muscle repair through expression of amphiregulin, an epidermal growth factor, *in vitro* and *in vivo* in mice (82). Tregs have been shown to have an amphiregulin-dependent role in tissue repair in the setting of infectious lung injury independent of their immune suppressive function (83). A role for Tregs in bone repair and regeneration has also been proposed to occur not only through the effect of Tregs on other immune cells to maintain bone homeostasis but also potentially through direct interactions with osteoblasts (15).

Various tumors contain Tregs, where their accumulation may suppress the immune response that would otherwise keep tumor cells at bay (84). CCL22 on tumors recruits CCR4^+^ Treg cells to the site and has been associated with tumor progression through their suppression of effector T cells that target tumor antigens (85, 86). Tregs constitutively express CTLA-4 and the benefit seen with CTLA-4 antibody therapy in cancer may in part be attributed to Treg depletion. Furthermore, CCL28 positive tumors can recruit Tregs where they have a proangiogenic role (87).

### Unstable Tregs

Several years ago, we demonstrated that Tregs were unstable in inflammatory tissues as they can lose FOXP3 and begin to turn on a broad array of potentially pathogenic pathways, such as the production of interferon-gamma and IL-17 (88). In part, this instability is due to decreased IL-2 signaling but may also reflect Treg inactivation through cytokines, such as IL-6. Purified IFNγ+ Tregs were suppressive *in vitro* but lacked Helios expression and were methylated at the Treg-specific demethylated region (TSDR) of FOXP3, characteristic of *in vitro*-induced Tregs. Thus, efforts to target Tregs in clinical settings will rely on efforts to repair and replace Tregs in autoimmunity and organ transplantation or destabilize and eliminate Tregs in the cancer and infectious disease settings.

## Human Therapies that Promote Tregs

### Rapamycin

While calcineurin inhibitors (such as Tacrolimus and Cyclosporine A) appear to inhibit Treg generation, rapamycin, which inhibits PI3K/AKT signaling through its direct interaction with the mTORC1 complex enhances Treg expansion and survival at the time it inhibits proliferation of Th1 and Th17 cells (89–102) (Figure 1). This is in part because PI3K/AKT signaling is a principal signaling pathway in Teff cells but less so in Tregs. In mice, rapamycin treatment leads to expansion of Tregs with increased suppressive activity *in vitro* and an enhanced ability to prevent pancreatic β cell transplant rejection *in vivo* (96). Administration of rapamycin in NOD mice, a model of T1DM, resulted in prevention of diabetes and restored tolerance to self-antigens due to expansion of Tregs (103). In patients with T1DM, rapamycin promoted expansion of Tregs (97) and enhanced their suppressive capacity (104). Again, unlike calcineurin inhibitors, rapamycin treatment maintained the proportion of Tregs in peripheral blood in renal transplant recipients (105) or even enhanced their frequency and reduced production of inflammatory cytokines, such as IL-1β, IL-6, IL-17, and IFNγ, in patients switched from tacrolimus to rapamycin (106). Similar effects are seen with everolimus, a synthetic derivative of rapamycin (107).

### Interleukin-2

The cytokine IL-2 plays a central role in Treg function and the balance between immunity and tolerance (46, 108, 109). Tregs express high-affinity CD25 and require IL-2 for survival. IL-2 interacts with the trimeric IL-2 receptor complex (CD122, CD132, and CD25) and signals primarily through the JAK/STAT pathway in Tregs to maintain FOXP3 expression and the development, proliferation, and suppressive function of Tregs (32, 47). IL-2 can expand Tregs *in vivo* and enhance their immune suppressive function (7, 110). The role of IL-2 in maintenance of self-tolerance is clear from studies of IL-2 or IL-2R-deficient mice that developed severe multi-organ autoimmune disease and early death (48–51). The levels of IL-2 and expression of CD25 on target cells influence the balance between immunity and tolerance. Tregs have a 10- to 20-fold lower activation threshold for IL-2 than effector T cells as assessed by the level of phosphorylated STAT5 (pSTAT5). The sensitivity to IL-2 in Tregs may be due to the function of the IL-2R signaling specificity: the MAPK, PI3K-AKT, and STAT5 pathways are all activated in effector T cells. In Tregs, the high PTEN expression may inhibit the PI3K signaling pathways and, therefore, activation relies on pSTAT5 signaling (65, 111, 112).

In autoimmune diseases, impaired IL-2 signaling is thought to affect the number and function of Tregs. The IL-2RA gene is one of the T1DM susceptibility genes (51, 52, 113–116). NOD mice treated with low-dose IL-2 showed increase in Tregs and reversal or prevention of diabetes (115, 117). In an EAE mouse model of multiple sclerosis, IL-2 treatment resulted in restoration of FOXP3 expression, Treg stability, and prevention of autoimmunity (118). However, depending on the dose, IL-2 can increase other potentially damaging leukocytes, including NK cells, activated CD8^+^ T cells and eosinophils [as a direct consequence of the activation of an innate lymphoid cell subset (ILC2)] (76).

Interleukin-2 was first used clinically to augment the immune response against tumor self-antigens in metastatic malignancies, such as melanoma and renal cell carcinomas, but variable clinical responses were seen with significant side effects (119, 120). Thus, in order to maximize the ability of IL-2 to selectively enhance Treg function, a series of mouse and human studies have been undertaken to examine IL-2 dosing as a means to shift the balance between immunity and tolerance in autoimmune diseases (111, 121, 122). IL-2 has been tested in clinical trials to treat graft versus host disease (GVHD), hepatitis C virus-induced vasculitis, and T1DM (108, 123–126) through a Treg-dependent pathway. A clinical response was shown in patients with GVHD enrolled in a Phase I, dose escalation, 8-week trial (123). Subjects exhibited not only an increased number of Tregs but also an increase in eosinophils and NK cells, which also express CD25. These investigators reported that IL-2 therapy increased Treg proliferation, thymic export, and enhanced resistance to apoptosis with minimal effects on conventional T cells (127). There was a selective effect of low concentrations of IL-2 on phosphorylation of STAT5, whereas IL-7 induced similar phosphorylation of STAT5 in conventional and Tregs at low concentrations. Patients with HCV vasculitis were treated with IL-2 in a Phase I/II clinical trial with the objective of increasing Tregs, which had been shown previously to correlate with successful treatment of this disease process. The majority of patients showed clinical improvement and there was an accompanying increase in the number of Tregs (124).

Preclinical studies by Rabinovitch et al. had demonstrated reversal of diabetes with combination of IL-2 and rapamycin (128). Thus, it was postulated that combining rapamycin with IL-2 would optimize inhibition of Teff signaling and concurrent augmentation of Treg signaling. Long et al. found that T1DM patients treated with rapamycin and IL-2 achieved increased frequency of circulating Tregs and sustained IL-2 signaling (125) but had transient worsening of β cell function as assessed by C-peptide following a mixed meal tolerance test. The trial was stopped after treatment of nine subjects. These investigators observed increased eosinophilia and natural killer cells. The relatively high doses of IL-2 (12 doses of 4.5 × 10^6^ IU) administered to the patients in that study was thought to account for the expansion of these potentially β cell toxic cells, although effects of the rapamycin could not be ruled out.

Thus, more recent efforts have been devoted to administering low-doses of IL-2 in patients with new onset diabetes. In a Phase I/II randomized double-blind placebo-controlled study, Hartemann et al. studied treatment with IL-2 at doses of 0.33, 1, or 3 million IU/day for a 5-day course. IL-2 induced a dose-dependent increase in the proportion of Tregs at all doses but at a dose of 1 × 10^6^ IU × 5, approximately one-tenth of the dose used in the trial of Long et al. Effects on Tregs predominated and there were insignificant changes in the proportion of NK cells in the peripheral blood. Adverse events also showed a dose–response with the most common AEs injection-site reactions and influenza-like syndrome (126). These studies were followed by Rosenzwajg et al. to determine the effects of IL-2 on induction of Tregs and NK cells in patients with T1DM (129, 130). They observed an increase in CD4^+^FOXP3^+^ and CD8^+^FOXP3^+^ Tregs, the proportion and duration of which was dose dependent. The Tregs expressed enhanced levels of activation markers and basal pSTAT5 and had a 20-fold higher sensitivity to IL-2 than Teff and NK cells. Global transcriptome analyses showed a dose-dependent decrease in immune response signatures. However, although they were able to induce a dose-dependent increase in Tregs, they did not observe a change in glucose metabolism (126).

Low-dose IL-2 is being trialed in other clinical settings, including rheumatoid arthritis, ankylosing spondylitis, systemic lupus erythematosus, psoriasis, Behcet’s disease, Wegener’s granulomatosis, Takayasu’s disease, Crohn’s disease, ulcerative colitis, autoimmune hepatitis, and sclerosing cholangitis (TRANSREG, ClinicalTrials.gov NCT01988506).

### CD3 Monoclonal Antibodies

In the 1990s, we established that CD3 monoclonal antibodies (mAbs) were shown to cause reversal of disease and induce immunologic tolerance in hyperglycemic NOD mice (131–135). To overcome the adverse events due to cytokine release associated with clinical use of Fc receptor (FcR)-binding CD3 mAb, molecules with mutations in the Fc region of the immunoglobulin were developed and further mechanistic studies in mice used either similarly modified molecules or F(ab’)2 fragments of the hamster anti-mouse CD3 mAb 145-2C11. Similar to the induction of Tregs following engagement of TCR with self-antigens, a relatively weak cognate signal may result in development of a regulatory phenotype rather than effector cells or depletion. The CD3 mAbs depleted effector T cells and caused a transient systemic rise in the percentage of CD4^+^FOXP3^+^ Tregs (136, 137). Expression of Helios was increased after anti-CD3 mAb treatment, suggesting that it increased the relative proportion of Tregs and stabilized their function (136). Belghith et al. described induction of adaptive TGFβ-dependent Tregs with anti-CD3 mAb even in CD28^−/−^ mice that lacked naturally occurring Tregs (137). These studies suggested that CD3 mAb induced pTregs from Tconv cells. Expanding on this notion, Esplugues et al. and Waldron-Lynch et al. showed in mice and in humanized mice that teplizumab, a non-FcR binding anti-CD3 mAb, caused migration of peripheral T cells to the lamina propria of the gut. At that location, there was induction of FOXP3 on T cells and Tregs. In the murine studies, this occurred following an inflammatory response heralded by the production of IL-17 and resolution with the formation of TGFβ and IL-10-producing cells. In the humanized mice treated with teplizumab, the gut migrating T cells expressed FOXP3 and produced IL-10. In the humanized mice, treatment with teplizumab prevented rejection of xenogeneic skin grafts (138, 139). Both CD4^+^ and CD8^+^ T cells were involved and the effects of the mAb required gut migration because it was blocked with the anti-α4 mAb, natalizumab. Interestingly, more recent studies by You et al. showed, in the setting of islet transplantation, that the effects of the anti-CD3 mAb were greatest after initiation of the graft-specific immune response (140). These findings were consistent with previous work in NOD mice and an experimental animal model of Multiple Sclerosis (EAE) showing that the efficacy was greatest at the time of peak immune response (131). This was confirmed in humans with T1DM where the greatest efficacy was observed at the time of clinical diagnosis (137).

Human trials with CD3 mAbs have shown effects and mechanisms consistent with induction of immune regulation (141–146). In general, an increase in the number of circulating CD4^+^CD25^+^FOXP3^+^ Tregs in the peripheral blood has not been seen in drug-treated patients with new onset T1DM. However, an increase in the number of circulating CD8^+^ T cells that have been suggested to have regulatory function has been consistently observed (147). These cells produce IL-10 family members and can suppress effector T cells *ex vivo*. Some evidence suggests that among these cells are CD8^+^ cells reactive with antigens from EBV, which may be reactivated with high doses of CD3 mAb (148). More recent studies have suggested that these cells show reduced expression of genes associated with T cell activation (149).

Mucosal, oral or nasal, administration of CD3 mAb has been shown to suppress autoimmunity in animal models of encephalomyelitis, collagen-induced arthritis, systemic lupus erythematosus, and diabetes (150–155). These studies demonstrated suppression of autoimmunity via induction of a Th3 type CD4^+^CD25^−^LAP (latency-associated peptide)^+^ Treg population. These Tregs are proposed to be a unique population given lack of CD25 expression and lower expression of FOXP3 that is not induced by mucosal anti-CD3 administration. Mucosal anti-CD3 seems to act primarily locally by inducing Tregs and not by reduction of effector T cells. Oral anti-CD3 has also been used in human studies to promote Tregs (156–158). Patients with non-alcoholic steatohepatitis treated with oral anti-CD3 in a Phase IIa trial showed that treatment was well tolerated and resulted in positive effects on hepatic and metabolic factors. Depending on treatment dose and time of analysis, they observed increased CD4^+^LAP^+^, CD4^+^CD25^+^LAP^+^, and CD4^+^CD25^+^FOXP3^+^ cells as well as increased TGF-β, supporting that oral anti-CD3 could induce Tregs in humans (156).

### Other Anti-T Cell Modulators

The major effect of anti-thymocyte globulin (ATG) and polyclonal anti-T cell antibodies was thought to involve broad elimination of T cells (159–164). However, subsequent *in vitro* and *in vivo* data suggest that ATG may actually selectively deplete Teff cells while sparing or in some cases even promoting the generation of Tregs (160, 165–171). The mechanisms that lead to depletion versus induction of regulatory cells have not been clearly defined.

Alefacept is a LFA3 fusion molecule that binds CD2 and results in eradication of CD2 expressing cells and has primarily been used in the treatment of psoriasis where it resulted in clinical benefit and sustained disease remission long after drug termination, suggesting lasting immune tolerance (172–174). In a randomized placebo-controlled, Phase II trial in T1DM preservation of C-peptide was improved with Alefacept vs. placebo treatment at 1 and 2 years. Phenotype studies of peripheral blood cells showed a decreased frequency of central memory and effector memory T cells and preserved Tregs resulting in increased Treg:Teff ratio similar to what was seen in mouse models with CD3 mAb (175–177).

## Cellular Therapy with Tregs

The ability to identify Tregs based on the expression of surface markers enabled investigators to isolate and potentially expand them *ex vivo* for cell therapy treatment. Moreover, the evidence that defects in Treg signaling that had been observed in patients might be repaired/reversed during culture provided even more support for adoptive cellular therapy with Tregs for treatment of autoimmune diseases. Methods to expand Tregs for clinical use were reported by our group using a 14-day expansion protocol with anti-CD3/28 and IL-2 (178). The expanded cells retained their immune suppressive function and had features consistent with nTregs, including high expression of CD25 and FOXP3 and demethylation of the TSDR. The Tregs could be expanded 500- to 2000-fold to as many as 3 × 10^9^ cells from 400 ml of peripheral blood. This represents more than 20% of the total estimated number of Tregs in humans (179).

Autoantigen-specific Tregs have had superior efficacy to polyclonal Tregs in preclinical studies but difficulties in expanding these cells and maintaining their phenotype and function led to the initial development of polyclonal Tregs for adoptive immune therapy (180, 181). An important consideration was that although Tregs require TCR-mediated activation to develop regulatory activity, their suppressive activity can spread within the affected tissues through bystander suppression and they can regulate local inflammatory responses through a combination of cell–cell contact and suppressive cytokine production. Thus, initial studies have been performed with polyclonal Tregs. In one study of 12 patients with new onset T1DM, aged 5–18 years, Marek-Trzonkowska et al. administered autologous Tregs that had been expanded with CD3/CD28 antibodies and IL-2. FOXP3 expression was found on >90% of the infused cells. A dose escalation of 10–30 × 10^6^ Tregs/kg in 1 or 2 doses was studied. After 1 year, 8/12 met the criteria of clinical remission (<0.5 U/kg/d of insulin) and two were insulin independent. They reported a significant improvement in glucagon-stimulated C-peptide responses and reduced insulin usage at 4 months and 1 year compared to untreated subjects. The infusions were well tolerated and antibody responses to a hepatitis B vaccine and rubella were apparently not affected by the treatment (182).

In a recently completed Phase I trial (NCT01210664), we observed that polyclonal FOXP3^+^CD4^+^CD25^+^CD127^lo^ Tregs could be efficiently isolated and expanded from patients with T1DM (183). This trial involved isolation and expansion of autologous Tregs over a 2-week culture period. The cells could then be shipped to a collaborating institution for infusion into patients, suggesting that development of this strategy for adoptive transfer of cells is feasible and need not be limited only to sites that are capable of performing the expansion on site. There were no significant safety signals from the study and the cells maintained their phenotype *in vivo*: there was no evidence of differentiation of the infused Tregs into other, potentially pathogenic phenotypes. Importantly, using an *in vitro* non-radioactive labeling technique (184), a subset of the adoptively transferred Tregs were observed to survive >1 year post infusion. A similar strategy is being used for treatment of patients with cutaneous lupus erythematosus (NCT02428309).

### Tregs in Organ Transplantation

Adoptive Treg therapy is being tested in transplantation settings. Tregs, partially matched for MHC, isolated from umbilical cord blood, have been used to treat graft-versus-host disease, associated with double umbilical cord blood transplantation. The Tregs were enriched from cryopreserved umbilical cord blood followed by an 18-day expansion culture with anti-CD3/CD28 beads and IL-2. Patients received a dose of 0.1–30 × 10^5^ Treg/kg after double umbilical cord blood transplantation. After infusion, the Tregs could be detected for 14 days. The Treg infusion was associated with reduced incidence of grades II–IV GVHD compared to historical controls with no deleterious effects on risk of infection, relapse, or early mortality (185). However, the Tregs were short lived, suggesting that either the third party cells were immunogenic or the absence of IL-2 *in vivo* in the BMT setting reduced Treg survival.

Solid organ transplants offer the opportunity to isolate, expand, and infuse antigen-specific Tregs from the graft recipient. In an ongoing trial (NCT02091232), Tregs are being isolated from mixed lymphocyte cultures in the setting of CTLA4Ig and infusing sorted Tregs from these cultures (The “ONE” Study). In patients with evidence of renal allograft rejection, another trial is expanding polyclonal Tregs for adoptive immune therapy (NCT02088931).

## Future Directions

Studies being conducted at UCSF and elsewhere have been initiated that utilize either polyclonal Tregs in other kidney and liver transplant settings with a goal of the discontinuation of immunosuppressive drugs (e.g., NCT02088931). These and other efforts will be essential in determining the potential use of *ex vivo* expanded cells in patients with these immune dysregulation diseases. Maximizing the functional activity, persistence, and migration of infused Tregs will be essential by either altering the Treg genetically or combining the treatment with growth factors, such as IL-2. Trials of Tregs plus low-dose IL-2 are ongoing (NCT1937468) and others are planned in T1DM. In an effort to increase the persistence of the cells *in vivo*, Parmer et al. showed that *ex vivo* fucosylation, which forms the sialyl Lewis X moiety on P-selectin glycoprotein ligand-1, improved *in vivo* persistence and had improved effects on a murine model of GVHD (186). We expect that additional biologics and antigen-based therapies will be introduced to protect and expand Tregs.

As mentioned above, antigen-specific Tregs are likely to be the best able to block unwanted immunity. In this regard, we have developed alloantigen-specific Tregs using a modified protocol that can selectively expand Tregs specific for MHC molecules expressed by donor tissue in kidney and liver transplant recipients. Donor-alloantigen-reactive Tregs (darTregs), isolated after leukapheresis, are being tested in patients undergoing liver transplantation at doses up to 800 × 10^6^ together with thymoglobulin, everolimus, mycophenylate mofetil (MMF), and solumedrol (NCT02188719). Future studies are likely to include Tregs, engineered to express TCRs, that are specific for auto or alloantigens or even antigens that are on or in the region of target organs to cause localization of the cells to the site of pathology and activation. Strategies used to introduce chimeric antigen receptors (CAR) for tumor antigens in effector T cells may be considered. As such, Ag-specific TCRs or chimeric antigen receptor-based genomic manipulation is now feasible with the use of genetic manipulation techniques, such as CRISPRCas9 to modify and enhance Treg specificity and function (187).

## Conclusion

Because of their central role in regulating immune tolerance and modulating immune responses, manipulation of Tregs represents a clear target for treatment of unwanted immune responses. Contrary to the strategy of immune suppression or cell deletion as a means to curtail responses, treatment with agents that enhance or cellular therapies with Tregs represent a strategy to restore immune tolerance. Trials that have enhanced the number and function of Tregs have shown success in treatment of a variety of conditions, and the development of technologies to expand Tregs and even select for antigen specific cells has created a new era for adoptive cellular immune therapies. Further refinements of the ways Tregs are selected and administered, including methods to improve their survival and maintain their efficacy, are likely to occur in the next several years.

## Author Contributions

AP, LC, JB, and KH reviewed the data and wrote the manuscript.

## Conflict of Interest Statement

Dr. Bluestone maintains agreements with MacroGenics Inc., Caladrius Biosciences, and Juno Therapeutics for development and testing of anti-CD3 mAb and regulatory T cells. The other co-authors declare that the research was conducted in the absence of any commercial or financial relationships that could be construed as a potential conflict of interest.
